# Special Issue: “Inflammatory Signaling Pathways Involved in Gastrointestinal Diseases”

**DOI:** 10.3390/ijms25021287

**Published:** 2024-01-20

**Authors:** Marianna Lauricella, Diana Di Liberto

**Affiliations:** Department of Biomedicine, Neurosciences and Advanced Diagnostics (BIND), Institute of Biochemistry, University of Palermo, 90127 Palermo, Italy

Inflammation is a defensive response of the innate and adaptive immune systems against injury and/or harmful microorganisms to restore homeostasis. The activation of inflammatory signaling pathways has been recognized in several gastrointestinal diseases, and signaling molecules that are implicated in these pathways are thought to be promising targets for new therapeutic strategies. 

In the light of these considerations, we have organized this Special Issue, titled “Inflammatory Signaling Pathways Involved in Gastrointestinal Diseases”, containing six papers (two research articles and four reviews).

Chronic inflammation of the gastrointestinal tract (GT) is a typical feature of Inflammatory Bowel Disease (IBD), a chronic idiopathic disease including Ulcerative Colitis (UC) and Crohn’s disease (CD) [1]. IBD’s etiopathogenesis involves many different factors, such as genetic predisposition, epigenetic variations, environmental factors, alterations in the intestinal microbiota, deregulation of the immune response, and chronic inflammation [2,3,4]. It is now well known that persistent oxidative stress and inflammatory responses leading to dysbiosis of the gut microbiota are associated with the onset and the progression of IBD [5]. Oxidative stress and gut dysbiosis, consisting of a decreased gut microbial diversity [6] with a higher presence of bacterial pathogenic species than beneficial species, are often induced by external stimuli, including a high-fat diet, smoking, disturbed circadian rhythms, and drug interventions. Over time, this leads to intestinal epithelium damage and increased intestinal permeability [7], with a consequent release into the blood of toxins, proinflammatory chemokines, and cytokines, such as tumor necrosis factor α (TNF-α), interleukin-1β (IL-1β), IL-6, and interferon-γ (IFN-γ) [5], as well as the activation of proinflammatory enzymes (e.g., iNOS, COX-2, and NOX) [8] and Mitogen-Activated Protein Kinases (MAPKs) signaling pathways [9]. As a result, a further increase in ROS levels in the GT [10] and a remodeling of the gut microbiota occur in a vicious cycle, in which dysbiosis and inflammation support each other, eventually causing a worsening of the inflammatory process that characterizes IBD patients [11].

Given the key role that is exerted by inflammation in the pathogenesis of IBD, the identification of new markers that are related to it represents a crucial aim in IBD research. In this scenario, the study by Scalia et al. [12] suggests a correlation between heat shock proteins (HSPs) and IBDs. HSPs are proteins that are expressed by the cells in stress conditions, whose genetic or acquired dysregulation has been connected to several pathological conditions called chaperonopathies [13,14]. The review highlighted that genetic and epigenetic deregulation of HSPs have been reported in IBD. However, the studies are controversial, because some of these deregulations may act as factors of susceptibility to the development of IBD, while others may act as protective factors assisting in the maintenance of the healthy/not inflamed intestinal tissue. Understanding these mechanisms is of fundamental importance to developing increasingly targeted therapies that consider molecular chaperones.

Another contribution, provided by Dvornikova et al. [15], outlines the main functions of histamine and its receptors (HRs) in the healthy and inflamed gut. Histamine sustains normal gut functioning and mediates pathogenic conditions by directly or indirectly modulating immune responses. The role of histamine and its signaling pathways in the inflammatory response makes it one of the main actors in the pathogenesis of IBD. In particular, the stimulation of H1R and H4R seems to be associated with proinflammatory effects, while H2R seems to mediate an anti-inflammatory effect in active IBD disease. However, the actual molecular mechanisms that underlie histamine/HRs-associated signaling pathways and are responsible for inducing inflammation in IBD are still unclear [16,17]. The authors also evaluated the experimental data from the literature from recent years, presenting a schematic overview of the main potential molecular signaling pathways that are associated with histamine receptors in IBD. The aim is that future research in this field could lead to the development of novel therapeutic approaches for IBD treatment based on agonists/antagonists of HRs or components of their signaling pathways.

The main goal of clinical treatment of IBD is to minimize the inflammatory response and heal the intestinal mucosal ulcers that characterize affected patients [18]. Currently, IBD treatment consists of immunosuppressants, aminosalicylic acid drugs, antibiotics, and monoclonal antibodies, such as anti-TNFα, with the aim of turning off the inflammation in the acute phase of the disease and/or extend remission periods [19]. However, long-term use of these drugs can lead to serious adverse reactions, poor clinical efficacy, and high recurrence rates and costs [20]. Collectively, novel therapeutic strategies for IBD treatment that are able to reduce oxidative stress and inflammation are urgently required.

Polyphenols are secondary metabolites of plants that are largely present in vegetables, fruits, nuts, and beverages such as coffee, tea, wine, and beer [21,22]. Due to their chemical structure, which is composed of multiple phenolic units, they can efficiently scavenge ROS, thus exerting an important antioxidant and anti-inflammatory activity both in vitro and in vivo [23,24,25,26]. A protective role for polyphenols against inflammation is making their use desirable in the treatment of IBDs [27]. The study by Pratelli and colleagues [28] highlights how polyphenols can restore gut dysbiosis and intestinal epithelium damage and reduce inflammation in IBD patients. Molecular mechanisms that are modulated by dietary polyphenols in IBD include the inhibition of NF-κB and the activation of Nrf2 transcriptional activity [29,30]. Furthermore, Pratelli et al. suggest that an “omics” approach could be useful for evaluating the individual response to nutrients and polyphenols that are introduced with diet, leading to a so-called “precision nutrition” research initiative that is associated with IBD management and clinical endpoint improvement. 

A new therapeutic strategy for IBD was also investigated by Scalavino and colleagues [31] in their article, where they analyzed the effect of miR-369-3p administration in IBD mice models. The authors demonstrated that miR-369-3p can reduce the expression of the immunoproteasome PSMB9 subunit and consequently modulate its catalytic activity, which is frequently increased in macrophages following their activation during immune responses to inflammatory and stress stimuli [32,33]. The immunoproteasome is a multi-catalytic protein complex that is expressed in all hematopoietic cells. An increased expression of immuno-subunits, followed by increased proteasome activities in activated macrophages, is frequently found in IBD patients, demonstrating its potential role in IBD’s pathogenesis [34,35,36]. Therefore, the identification of molecules that are able to inhibit the activities of this complex is widely attempted, as it is a future alternative therapeutic approach for the treatment of inflammatory diseases, such as IBD [37,38,39].

Disorders involving gut–brain interactions (DGBIs), formerly called functional gastrointestinal disorders (FGIDs), include irritable bowel syndrome (IBS) and functional dyspepsia (FD). The pathophysiology of DGBIs is complex and includes a combination of motility disturbance, visceral hypersensitivity, altered mucosal and immune function, altered gut microbiota, and altered central nervous system processing. Immune system deregulation and inflammation and a compromised barrier function have been correlated with DGBIs and represent risk factors for the development of IBD [40,41,42]. Due to the complex etiology of the problem, the available treatment methods do not completely cure the disease. The study by Szadkowska et al. [43] discusses the beneficial effect of *Cirsium palustre* on gut disorders. *Cirsium palustre* is an herbaceous plant belonging to the Asteraceae family, which displays anti-inflammatory and antimicrobial effects due to the presence of flavonoids. The authors demonstrated that selected preparations and flavonoids from *C. palustre* have a pronounced motility-regulating effect on the swine colon, as well as proven beneficial antioxidant and antibacterial effects in this ex vivo system, suggesting the need for further investigations into their use in disorders involving DGBI patients’ treatment [44,45,46]. 

Long-standing intestinal inflammation in the GT, leading to the disruption of the normal intestinal structure and to the dysregulation of the intestinal mucosal immune system [47], can also predispose to cancer. 

Barrett’s esophagus (BE) is a premalignant lesion involving the development of intestinal metaplasia in the distal esophagus. This represents an adaptational mechanism in the distal esophagus, which is caused predominantly by a long-term exposure to bile reflux. Some recent investigations by Maslenkina et al. [48] described the role of NOTCH, hedgehog (Hh), NF-κB, and IL6/STAT3 signaling in the pathogenesis of Barrett’s esophagus (BE). The study also focused on catastrophic genetic events due to impaired DNA reparation [49,50,51], the rapid activation and amplification of oncogenes such as MYC and MDM2 oncogenes, and the inactivation of tumor suppressor genes [52] following repeated reflux exposure and leading to the formation of dysplasia and esophageal adenocarcinoma (EAC) [53,54,55,56]. Their data provide evidence that genomic catastrophe is important in malignant transformations in BE, thus serving as an alternative mechanism of carcinogenesis and a “point of no return” after which malignant transformation becomes inevitable [57].

We would like to share our gratitude to all the authors who submitted their outstanding research to this Special Issue. Their manuscripts highlighted some of the molecular mechanisms underlying inflammation that characterize different gastrointestinal diseases and compounds with potential antioxidant action for application as adjuvant molecules in the treatment of these diseases. 
